# Characterization of Folding Cores in the Cyclophilin A-Cyclosporin A Complex

**DOI:** 10.1016/j.bpj.2015.02.017

**Published:** 2015-04-07

**Authors:** Jack W. Heal, Stephen A. Wells, Claudia A. Blindauer, Robert B. Freedman, Rudolf A. Römer

**Affiliations:** 1 MOAC Doctoral Training Centre, University of Warwick, Coventry, United Kingdom; 2 Institute for Advanced Study, University of Warwick, Coventry, United Kingdom; 3 Centre for Scientific Computing, University of Warwick, Coventry, United Kingdom; 4 Department of Chemistry, University of Warwick, Coventry, United Kingdom; 5 Department of Physics, University of Warwick, Coventry, United Kingdom; 6 School of Life Sciences, University of Warwick, Coventry, United Kingdom; 7 Department of Chemistry, University of Bath, Bath, United Kingdom

## Abstract

Determining the folding core of a protein yields information about its folding process and dynamics. The experimental procedures for identifying the amino acids that make up the folding core include hydrogen-deuterium exchange and Φ-value analysis and can be expensive and time consuming. Because of this, there is a desire to improve upon existing methods for determining protein folding cores theoretically. We have obtained HDX data for the complex of cyclophilin A with the immunosuppressant cyclosporin A. We compare these data, as well as literature values for uncomplexed cyclophilin A, to theoretical predictions using a combination of rigidity analysis and coarse-grained simulations of protein motion. We find that in this case, the most specific prediction of folding cores comes from a combined approach that models the rigidity of the protein using the first software suite and the dynamics of the protein using the froda tool.

## Introduction

The protein-folding problem has been a prevalent issue for the past 50 years, as the emerging protein structure crucially determines flexibility, mobility, and, ultimately, function (1). The two principal competing theories on how protein folding initiates are diffusion-collision (2) and nucleation-condensation (3). Indeed, it may well be that both are valid depending on which protein is being investigated (4). It can be intuited that residues that “collapse early during folding” (5) might be particularly important to the overall folding process and are usually referred to as defining a folding core. However, this set of residues is difficult to ascertain precisely. One way to define a folding core experimentally is through Φ-value analysis (6). This approach focuses on the folding process by using point mutations to determine the impact of particular residues on the energy of the transition state in a one-step folding process. An alternative is to study the dynamics of the folded structure through hydrogen-deuterium exchange (HDX) NMR experiments. For the examples of barnase and chymotrypsin inhibitor 2, it has been shown that the two definitions are consistent in that slowly exchanging residues in HDX have high Φ-values (7).

Establishing folding cores through Φ-value analysis or HDX provides valuable insight into protein folding and dynamics but also involves extensive experimental work. For this reason, the prediction of HDX folding cores through rapid computational methods is of ongoing interest (4,8–12). One method for predicting HDX folding cores uses rigidity analysis and is implemented in the first software package (4,8). The method makes inferences about protein flexibility based solely upon the static crystal input structure. Surface exposure of the exchanging protons and protein motion, known to be important for HDX measurements and hence for folding cores (5,7), are ignored in this method. Here, we seek to improve upon the predictive power of rigidity analysis for folding-core prediction by incorporating missing information on surface exposure and dynamics of the protein.

**Figure 1:**
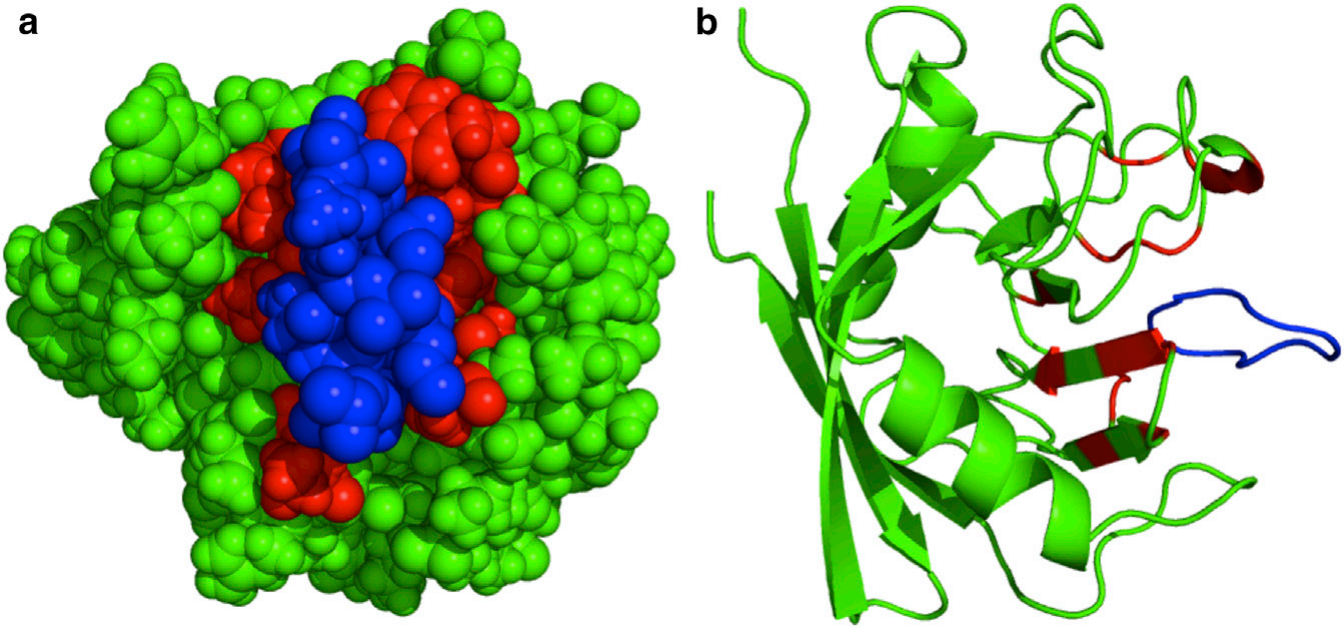
Sphere (*a*) and cartoon representation (*b*) of the CypA-CsA complex and the CypA binding site (using PDB structure 1CWA and the PyMOL visualizer (17)). CsA is indicated in blue and CypA in green. The 15 residues of CypA that have a heavy (nonhydrogen) atom within 4 Å of the atoms in CsA are red. To see this figure in color, go online.

We have selected cyclophilin A (CypA), a multifunctional 18 kDa protein with 165 residues, as the basis protein for our study, since it is large enough to exhibit complex folding behavior and at the same time readily investigated by HDX. It is known to bind strongly to the immunosuppressant drug cyclosporin A (CsA) (13–15). The structure of the CypA-CsA complex is shown in Fig. 1 with the binding-site residues highlighted (16).

In our experiment, we study the HDX behavior of unbound CypA and also its complex with CsA. Using these HDX data, we establish the resulting folding cores. Having found the HDX folding core in our experiments, we next apply first to unbound CypA and to the CypA-CsA complex, establishing the theoretical first folding core (FIR) in each case. We compare the resulting predicted folding cores with the HDX results and show that the established theoretical method of rigidity analysis implemented using the first software provides a reasonable prediction of the experimental folding core (4,8,18). Nevertheless, although the first method is impressive, it is not perfect in that it does not capture the changes in the HDX folding core observed upon ligand binding. Indeed, the predicted folding core is larger in the absence of the ligand, a result in contrast with what is observed experimentally.

We then change the hydrogen bond (HB) and hydrophobic tether (HP) networks so that these interactions are absent for surface atoms. With this modification, we enhance the flexibility of the protein surface so that the remaining rigid residues can better correlate with the experimental folding core. Building upon this, we model the prospective dynamics of the protein using coarse-grained simulations. By applying these techniques, we are able to probe the propensity for large-amplitude motions that can only be accessed over long timescales such as those of our HDX experiments. We show that our theoretical approach, which combines rigidity-analysis information from coarse-grained simulations and surface exposure, markedly improves the correlation between theory and experiment.

## Materials and Methods

### The CypA-CsA complex

The multifunctional CypA belongs to the large class of ligand-binding proteins. CypA acts as a peptidyl-prolyl *cis-trans* isomerase in addition to performing other roles when binding to different molecules, such as the HIV-1 capsid protein (19–21). The CypA-CsA complex is strongly bound (13–15) with dissociation constant *K*_D_ = 46 nM (22). Most commonly used to suppress organ rejection after a transplant, CsA has also been administered to treat ulcerative colitis, cardiac disease, and a number of autoimmune diseases (23–25). It is the CypA-CsA complex that binds to and inhibits the T-cell activator calcineurin (CN) and thus has an immunosuppressant effect (26,27). Details of the expression and purification process and the characterization of the CypA-CsA complex are given in the Supporting Material.

HDX experiments have been conducted previously on unbound CypA (28) but not yet on the CypA-CsA complex. NMR has been used to solve the structure of CypA (15), as well as that of the CypA-CsA complex (13,14). Our HDX results broadly agree with the data on unbound CypA and in addition elucidate the effect of ligand binding in the CypA-CsA complex.

### Rigidity analysis with first

Protein rigidity analysis is a computational method that rapidly identifies rigid and flexible regions in a protein crystal structure (29,30). The structure is considered as a molecular framework in which bond lengths and angles are fixed while dihedral angles are permitted to vary. Atomic degrees of freedom are then matched against bonding constraints (30–36). Covalent bonds, polar interactions (including hydrogen bonds and salt bridges), and hydrophobic tethers can all be included as bonding constraints. The output of the algorithm is a division of the structure into rigid clusters and flexible regions, known as a rigid-cluster decomposition (RCD). The RCD clearly depends on the constraints present in the bond network, the strength and location of which are determined solely from the geometry of the input structure. A systematic removal of hydrogen bonds from weakest to strongest leads to a loss in rigidity that we can relate to the unfolding of the protein (29). This is referred to as a rigidity dilution (RD). The pattern of rigidity loss can be used to gain insight into structural and functional properties of the protein (29,37,38). Indeed, RDs have been used previously to predict the HDX folding core for a number of proteins (4,8), not including CypA.

The Protein Data Bank (PDB) (39) x-ray crystal structure 1CWA of the CypA-CsA complex (40) was used for all simulations of protein rigidity and motion. After removing crystal water molecules, Reduce software (41) was used to add the hydrogen atoms and to flip side chains of Asn, Gln, and His residues where necessary. For simulations of the unbound protein, CsA was deleted from the structure manually using PyMOL (17), which was used for all molecular visualization. We note that the unbound structure for CypA is highly similar to the bound structure. Indeed, the backbone of 1CWA aligns with a different structure from that of the unbound protein (1W8V) with an RMSD of 0.26 Å.

The strength of each hydrogen bond, measured in kcal/mol, was calculated as a function of the geometry of the donor, hydrogen, and acceptor atoms using the distance- and angle-dependent Mayo potential (37,42). Only hydrogen bonds with a bond energy more negative than the energy cutoff parameter, *E*_cut_, are included in the network. During an RD, *E*_cut_ is lowered, causing some hydrogen bonds to be excluded from the network. Rigidity dilution involves systematically lowering *E*_cut_ and reevaluating the RCD. Rigidity analysis was conducted using first version 6.1 (43).

### A modified bond network

We distinguish between buried and exposed residues in the protein by drawing points on the surface of a sphere around each atom, with radius equal to the van der Waals’ radius of the atom plus that of a water molecule (1.4 Å). If the points of this sphere do not contact those of neighboring atoms, then the atom is labeled as being exposed. An atom is assigned a burial distance *R* = 0 Å if it is exposed, and *R* = *k* Å otherwise, where *k* is the smallest distance to an exposed atom (see the Supporting Material for details).

We modify the standard bond network defined by first based on the surface exposure of atoms with HBs and HPs. By demanding that both interacting atoms in an HB or HP constraint are buried within the protein, i.e., not exposed on the surface, we enhance the flexibility of the protein surface. The folding core generated in this way consists of residues that are both rigid and buried within the protein.

### Coarse-grained mobility simulation

The coarse-grained elastic network model implemented using the ElNemo software (44) allows for a rapid and accurate calculation of the normal modes of motion (45,46). The software froda allows rapid simulation of protein motion along these normal-mode vectors to generate new conformations satisfying the constraints of the first bond network (37,47).

The 10 lowest-frequency nontrivial normal-mode vectors, **m**_7_–**m**_16_, capture well the large-scale motion of a protein (48). We use froda to generate 2000 conformers of the protein along both trajectories (parallel and antiparallel) defined by each of the vectors **m**_7_–**m**_16_. To generate a new conformer, we force each atom to move a distance of 0.01 Å in the direction specified by the normal-mode vector, as well as a distance of 0.01 Å in a random direction. We then check for steric clashes and impose the bond network determined by first with *E*_cut_ = −2.0 kcal/mol, demanding that the bonds in this network are satisfied in terms of their distance and angle. We repeated these simulations at five different *E*_cut_ values (−0.5, −1.0, −1.5, −2.0, and −3.0 kcal/mol) and found that the nature of the results is not particularly sensitive to changes in this value (data not shown; see Heal (49)).

### Quantitative measures for comparing folding cores

Let *N*_Ex_ and *N*_Th_ be the numbers of residues contained in an experimentally and theoretically determined folding core, respectively. Clearly, *N*_Ex_ = *N*_Th_ is a necessary condition for agreement of experimentally and theoretically estimated folding cores. However, it is not just the number of residues, but of course the agreement of the specific set of residues in both experimental and theoretical folding cores, that is is most important. To capture this, let us define T as the number of residues correctly identified by a theoretical prediction of the experimental folding core, i.e., T is the number of true positives. For a perfect agreement, we have $T=N_{\text{Th}}$ while the expected T attained randomly is *N*_Th_ *N*_Ex_ / *N*. Here, *N* denotes the total number of amino acids in the protein (8). We can define the specificity, *α*, and sensitivity, *γ*, of a theoretical folding core prediction as

(1) $$\alpha =\frac{T}{N_{\text{Th}}}$$

and

(2) $$\gamma =\frac{T}{N_{\text{Ex}}}.$$

The specificity, *α*, measures the fraction of residues identified by the theoretical method that are also part of the experimental folding core. The sensitivity, *γ*, shows the proportion of residues in the experimental core that have been correctly predicted by the theoretical method. A perfect correspondence between theory and experiment, $T=N_{\text{Th}}=N_{\text{Ex}}$, would yield *α* = *γ* = 1, whereas for a completely wrong identification, T=0, we have *α* = *γ* = 0.

Another, previously defined, quantitative measure (8) is the so-called folding-core identification enhancement factor, *ϵ*. This is the ratio of T to the number of residues expected to be identified by a random selection,

(3) $$\epsilon =\frac{TN}{N_{\text{Th}}N_{\text{Ex}}}.$$

A theoretical method with random probability of success has ϵ=1, and when ϵ>1, the match is better than random. We shall also compare our *α* and *γ* measures to *ϵ*.

### Experimental procedure

The following is a brief outline of the experimental procedure. Full details of protein expression, purification, biophysical characterization, NMR spectrum assignments, and HDX experiments are presented in the Supporting Material. The protocol for protein expression and purification was adapted from Liu et al. (22). Circular dichroism was used to determine the folded state of the protein and fluorescence spectroscopy to demonstrate CypA-CsA complex formation (50,51). NMR assignments were determined from a series of 3D (^1^H,^15^N,^1^H) total correlation spectroscopy-heteronuclear single quantum coherence (TOCSY-HSQC) and nuclear Overhauser effect spectroscopy-heteronuclear single quantum coherence (NOESY-HSQC). For the HDX experiments, lyophilized protein was resuspended in phosphate buffer at pH 6.5 prepared with D_2_O. Exchange was monitored through a series of two-dimensional [^1^H,^15^N] HSQC spectra acquired on a Bruker AV II 700 spectrometer.

## Results and Discussion

### The first folding cores

We simulated RDs by systematically lowering *E*_cut_, i.e., removing the hydrogen bonds in order of strength from weakest to strongest (37). We used first to generate an RCD each time a hydrogen bond was removed. In a one-dimensional representation of an RCD, each residue in the primary structure is labeled as being rigid or flexible depending on the rigidity of its C_*α*_ atom. We show rigid residues as blocks colored according to their rigid-cluster membership.

**Figure 2:**
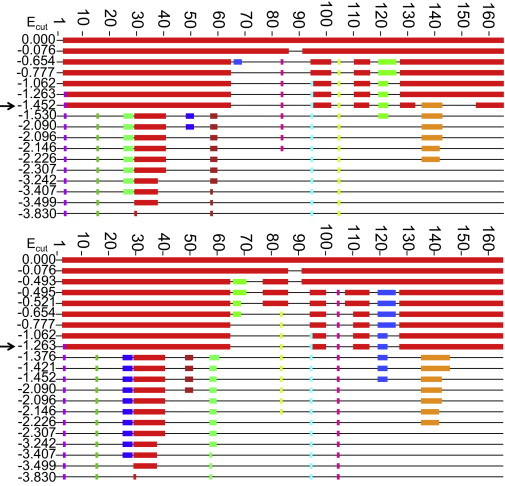
RD plots of the CypA-CsA complex (*upper*) and unbound CypA (*lower*). The RCD is shown at different values of *E*_cut_ (kcal/mol). Rigid residues are shown as thick colored blocks and flexible regions as thin horizontal black lines. Residues that are mutually rigid are shown in the same color. The line representing the first folding core in each case is indicated by an arrow. To see this figure in color, go online.

We visualize the pattern of rigidity loss during RDs by plotting the one-dimensional representation of the RCD each time this changes. Such plots for the CypA-CsA complex and the unbound CypA are given in Fig. 2. When $\left|E_{\text{cut}}\right|$ is small, the protein is largely rigid and many of the residues are represented as blocks. As *E*_cut_ becomes more negative, i.e., as stronger bonds are excluded from the bond network, more residues become flexible.

In both of the RD plots, there is a clear and abrupt transition from the largely rigid state to the largely flexible state, consistent with the first-order rigidity loss expected for a predominantly *β*-sheet protein (37). The lowest line in the RD plot, where at least three residues of two or more secondary structures (as determined using the DSSP algorithm (52)) are part of the same rigid cluster, determines the first folding core (4,8,18,53). We refer to the *E*_cut_ corresponding to this line as the folding-core energy, *E*_fc_. For unbound CypA, *E*_fc_ = −1.263 kcal/mol, and for the CypA-CsA complex, *E*_fc_ = −1.452 kcal/mol. That the CypA-CsA complex has a lower *E*_fc_ suggests that ligand binding confers stability on the complex, as more bonds need to be broken to render the protein mostly flexible (53). The residues that are mutually rigid in the RCD evaluated at the *E*_fc_ (Fig. 2, *red*), form the first folding core.

### The HDX folding cores

**Figure 3:**
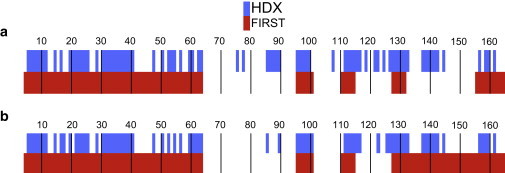
The HDX folding cores (*blue*) are given along with the first folding cores (*red*) for the CypA-CsA complex (*a*) and unbound CypA (*b*). The residue numbers along the protein backbone are indicated above each section, with thin vertical lines added every 10 residues for clarity. Only the residues that are part of the folding cores are colored. To see this figure in color, go online.

We define the set of residues for which a corresponding backbone amide signal remains in the HSQC spectrum after 110 min as the experimentally determined HDX folding core of the complex. These residues are listed in Table 1 and are also indicated in Fig. 3.

The published HDX experiments on unbound CypA resulted in a classification of CypA residues in terms of their exchange rates, *k*_ex_ (28). Twelve residues, including the proline residues, were not categorized, since they were not identified in the HSQC spectrum. We also carried out HDX experiments on the unbound protein, and our data, shown in the Supporting Material, were in agreement with the previously published result (28).

Here, we have drawn on the exchange rates determined in Shi et al. (28) and defined the residues with *k*_ex_ < 10^−2^ min^−1^ as the folding core of the unbound protein, a definition congruent with that applied to our data set for the CypA-CsA complex (see above). According to this approach, the HDX folding core for unbound CypA has 73 residues.

### Comparison of first and HDX folding cores

In Fig. 3, we compare the HDX and first folding cores along the primary structure of the CypA-CsA complex and unbound CypA. Residues that form part of the folding cores are represented as colored blocks. In both cases, the first folding cores largely overlap with the HDX folding cores. The small changes that do occur upon ligand binding in the HDX folding cores are not very well captured in the two first folding cores. Rather, these differ only between residues 133 and 155, whereas the HDX folding cores remain largely unaffected in this region. Notably, the first folding core for the CypA-CsA complex is smaller than that for the unbound protein. This contrasts with both our expectation and experimental finding as given above, where we show that the HDX folding core increases in size upon ligand binding. It highlights a problem with a theoretical method that only uses first. Ligand binding to the surface of CypA causes the binding-site residues to become buried where before they were exposed, which may affect their HDX exchange rates. This effect is not modeled in first, where we merely consider the hydrogen-bond network of the initial, static crystal structure.

We find that the HDX folding core does not change dramatically upon ligand binding. The 10 residues in bold in Table 1 are slowly exchanging in the presence of the CsA ligand but not part of the HDX folding core for unbound CypA. Eight of these belong to flexible regions proximal to the binding site. These changes suggest shielding or constraining of the unstructured regions near the binding site in the presence of the ligand. The small increase in folding-core size, from 73 to 80 residues, is consistent with the expectation that the presence of a ligand is likely to shield certain residues from the surface of the protein. There are also three assigned residues—G18, A101, and I158—that are, surprisingly, part of the unbound folding core according to Shi et al. (28) but not part of the bound folding core.

### Folding cores FIR_B_, SeS, FRO, and F+F

We now introduce four additional theoretical folding cores, which we consider alongside the First folding core in our subsequent analysis. We refer to these folding cores as FIR_B_, SeS, FRO, and F+F. FIR_B_ is similar to the first folding core, but with the additional condition that a residue must be buried as well as rigid to be part of FIR_B_. This condition was imposed through the modified bond network in First, which demands that both partners in an HB or HP network are buried.

For SeS, we take the secondary structural units (*α*-helix and *β*-strand) as determined using the DSSP algorithm. This is our simplest theoretical predictor of a folding core based only on secondary structure. The results of our coarse-grained simulations of protein motion are incorporated in FRO. Here, the final (2000th) conformer from each of our 20 simulations was subjected to a burial-distance analysis, and those residues that were buried in >10 of these conformers were included in FRO. The burial distance, *R*, of each residue was determined from its amide nitrogen (see the Supporting Material), and if *R* > 1.5 Å, the residue was classed as buried. We emphasize that the results of this mobility-based analysis depend indirectly on the results of the rigidity analysis; first identifies a constraint network and froda then explores the motion that is possible within those constraints. Therefore, it is possible for a residue to be 1) rigid but exposed, thus lying in FIR or FIR_B_ but not in FRO; or 2) nonrigid but well protected by burial within the protein, thus lying in FRO but not in FIR or FIR_B_. This suggests that to predict the results of the HDX folding core, which depend upon surface exposure as well as dynamics (inherited from flexibility), a combination of the approaches employed by first and froda may be necessary. F+F is simply the intersection of residues in FIR and FRO.

**Figure 4:**
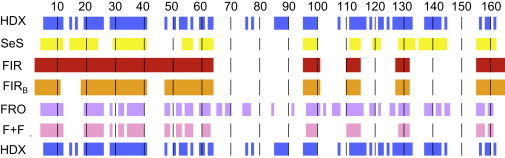
Comparison of the experimentally determined HDX folding core for CypA-CsA (*blue*; shown both above and below the other folding cores), with the five folding cores computed using the theoretical approaches outlined in the text and labeled accordingly (from top to bottom, SeS (*yellow*), FIR (*red*), FIR_B_ (*orange*), FRO (*purple*), and F+F (*pink*)). In each case, the folding-core residues in the primary structure of CypA are represented as colored blocks and the nonfolding core residues are white. Residue numbers along the protein backbone are indicated above the table body, with thin black vertical lines added every 10 residues for clarity. To see this figure in color, go online.

In Fig. 4, each of the five theoretical folding cores is compared with the HDX folding core for the CypA-CsA complex. In each case, folding-core residues along the protein backbone are shown as bold blocks of color.

**Figure 5:**
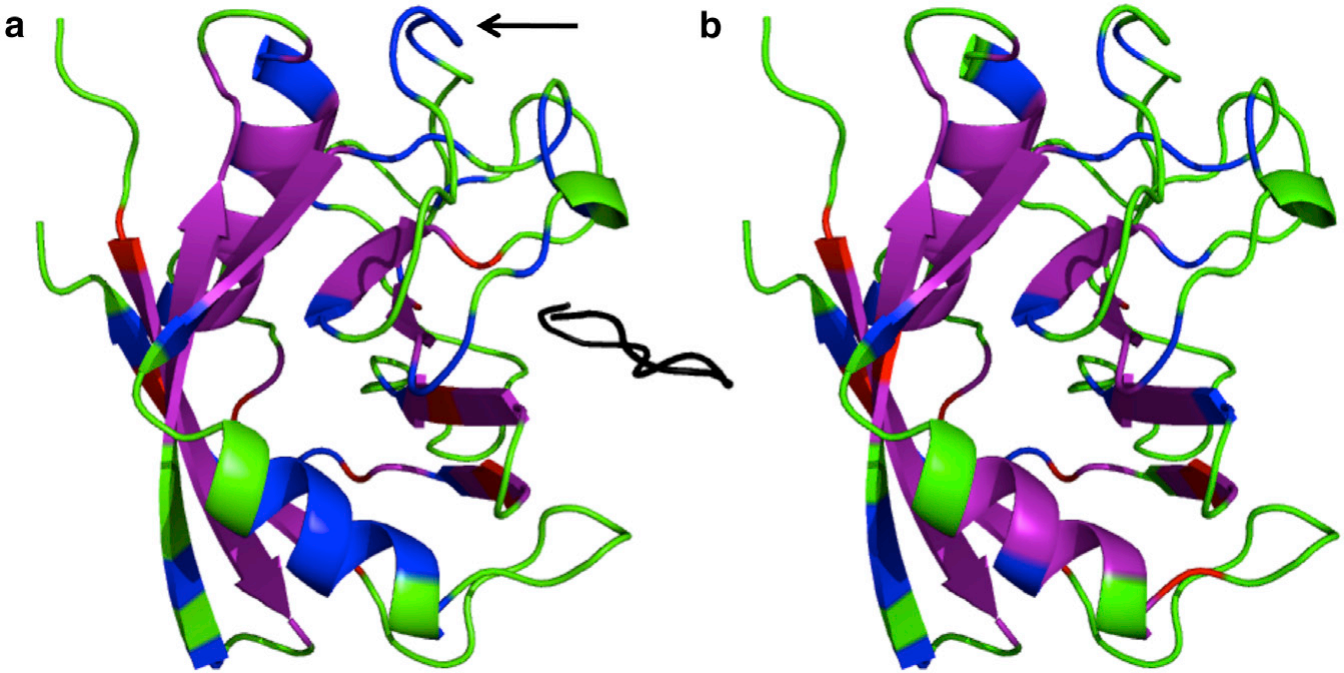
Cartoon representation of a comparison of HDX folding cores with F+F folding cores for the CypA-CsA complex (*a*) and unbound CypA (*b*). In both cases, residues that are part of both folding cores are purple, those found only in the HDX folding core are blue, those in the F+F folding core only are red, and those that are not part of either folding core are red. CsA is colored black in (*a*). The arrow in (*a*) indicates residues 86–90. To see this figure in color, go online.

We find that SeS consists almost entirely of slowly exchanging amino acids, although there are also many slowly exchanging residues that are not part of a secondary structure unit. As expected, FIR_B_ is smaller than FIR, since there are fewer constraints present throughout the rigidity dilution. The surface-exposed residues 12–18 and 41–47 are part of FIR but not part of FIR_B_. The residues in FRO are also for the most part slowly exchanging. The combined F+F folding core matches the HDX core with high specificity. Fig. 5 presents a graphical comparison between the HDX folding core and F+F on the structure of CypA. We observe that in the absence of the ligand, residues 86–90 are mostly absent from both theoretical and experimental folding cores. The relatively small number of false positives (Fig. 5, *red*: 10 residues in (*a*) and 8 in (*b*)) relative to false negatives (Fig. 5, *blue*: 36 residues in (*a*) and 22 in (*b*)) demonstrate the high ratio of specificity to sensitivity for this method.

We note that some parts of the HDX folding core, for example, residues 86–90, are poorly predicted by all of the methods. This region corresponds to a flexible, surface-exposed, unstructured region in the crystal structure (Fig. 5 *a*, *arrow*). Our methods, based around predicting rigid, buried, and immobile regions, fall short of identifying this area as slowly exchanging.

### Quantitative analysis of theoretical folding cores

Table 2 shows the number of residues in each theoretical folding core, along with the number of true positives, T. The comparison pictured in Fig. 4 is made quantitative with the specificity, *α*, sensitivity, *γ*, and enhancement factor, *ϵ*, as defined in Eqs. 1–3, respectively.

The *ϵ* values of the five theoretical folding cores, the lowest of which is 1.33 (for the previously established first folding core FIR), demonstrate improvement over random selection. F+F scores highest for both the CypA-CsA complex and the unbound CypA, suggesting that the combined approach has merit. This method is highly specific (*α* ≥ 0.8 in both cases), although its low *γ* scores show that it does not capture enough of the HDX folding core to successfully capture the impact of ligand binding on HDX. SeS is highly specific (*α* ≥ 0.77) and sensitive (*γ* ≥ 0.74), although this method of folding-core selection is clearly inappropriate for predicting changes upon ligand binding, since it does not change. The two folding cores derived solely using first (FIR and FIR_B_) do not reflect the experimentally observed increase in folding-core size upon ligand binding. Only when dynamics are also incorporated, i.e., in FRO and F+F, is this qualitative effect reflected.

We have also estimated the variation in *α* and *γ* in Table 2, assuming a ±5% error in *N*_Ex_, *N*_Th_, and T. The six proline residues in CypA do not appear in the ^1^H,^15^N HSQC spectrum due to their lack of an amide proton. For this reason, HDX experiments cannot inform on whether prolines are part of a folding core. We therefore also calculated *ϵ* with *N* = 159. The ordering of the theoretical methods remains the same, and coupled with the small variation in *α* and *γ*, this shows that our quantitative comparison is robust.

## Conclusions

The experimental HDX folding core for the CypA-CsA complex is highly similar to that for the unbound protein, albeit with a small number of additional residues. This small change is consistent with previous observations of only subtle conformational change in CypA upon ligand binding (15,54). The first folding cores differ more substantially. Ligand binding confers rigidity upon the structure but alters the pattern of rigidity loss so that the first folding core in fact decreases in size. In both cases, the first folding core is a reasonable match for the HDX folding core, in agreement with previous folding-core predictions using first (4). The first folding core is defined by the RCD at *E*_fc_, a value that decreases upon ligand binding. This means that when the folding cores are compared for the protein before and after ligand removal, we are comparing RCDs at different *E*_cut_ values. As a result, there may be more constraints present in the *E*_cut_ value for the unbound protein than in that for the complex, resulting in the unlikely prediction of a larger folding core in the absence of a ligand. first is a tool that can be implemented rapidly, and this study complements previous studies demonstrating its utility for folding-core prediction (4,8,18,53). Nevertheless, our work also shows that the first-based folding-core predictions are not yet accurate or sensitive enough to capture the impact of ligand binding for CypA. For the purpose of predicting subtle effects of ligand binding, simply analyzing patterns of rigidity, as done through first, appears insufficient. The trade-off between rapid computation on the one hand, as achieved with first, and the necessary accuracy of folding core prediction on the other hand needs to be more finely balanced.

As a way forward, we have introduced and discussed rapid computational methods for adding information on surface exposure and protein dynamics into a rigidity-based folding core definition. These improve markedly upon the first folding core for predicting the HDX folding cores of both CypA-CsA and unliganded CypA. In addition, we show that they are less sensitive to the erroneous increase in folding-core size upon ligand removal observed using first with the standard bond network. On balance, F+F, which combines rigidity and motion, appears to be the best choice for a computational determination of the folding core from a protein’s structural information alone. Nevertheless, no method achieves a perfect score of *α* = *γ* = 1, either for the uncomplexed CypA or for the CypA-CsA complex. This insensitivity to ligand binding is disappointing but unsurprising due to the scale of the challenge, both for CypA-CsA and in general.

## Figures and Tables

**Table 1:** Residues of the HDX folding core for the CypA-CsA complex

CypA-CsA HDX folding-core residues
V6, F7, F8, D9, I10, A11, V12, E15, L17, V20, **S21**, F22, E23, L24, F25, A26, V29, K31, T32, A33, E34, N35, F36, R37, A38, L39, S40, T41, Y48, S51, F53, **H54**, R55, I57, F60, M61, Q63, G64, **K76**, **I78**, E86, **N87**, **F88**, **I89**, L90, G96, I97, L98, S99, M100, **N108**, F112, F113, I114, C115, T116, A117, **T119**, **L122**, D123, K125, V127, V128, F129, G130, K131, V132, K133, I138, V139, E140, A141, M142, E143, F145, T157, A159, D160, G162

Residues in bold print are slowly exchanging only in the presence of the ligand.

**Table 2:** Quantitative measures of agreement between theoretical folding cores and the experimental HDX folding core

Folding core	*N*_Ex_	*N*_Th_	T	*α*	*γ*	*∈*_165_	*∈*_159_
CypA-CsA							
SeS	80	75	59	0.79 ± 0.04	**0.74** ± 0.04	1.62	1.56
FIR	80	86	57	0.66 ± 0.04	0.71 ± 0.04	1.37	1.32
FIR_*B*_	80	72	53	0.74 ± 0.04	0.66 ± 0.04	1.52	1.46
FRO	80	80	56	0.70 ± 0.04	0.70 ± 0.04	1.44	1.39
F+F	80	54	44	**0.81** ± 0.05	0.55 ± 0.03	**1.68**	**1.62**
Unbound CypA							
SeS	73	75	58	0.77 ± 0.04	0.79 ± 0.04	1.75	1.68
FIR	73	112	66	0.59 ± 0.03	**0.90** ± 0.05	1.33	1.28
FIR_*B*_	73	72	52	0.72 ± 0.04	0.71 ± 0.04	1.63	1.57
FRO	73	76	54	0.71 ± 0.04	0.74 ± 0.04	1.61	1.54
F+F	73	59	51	**0.86** ± 0.05	0.70 ± 0.04	**1.95**	**1.88**

Values are for CypA-CsA complex (*upper*) and unbound CypA (*lower*), with *N* = 165. Bold numbers indicate the largest selectivity, *α*, sensitivity, *γ*, and enhancement, *∈*, for CypA-CsA and unbound CypA. The error estimates for *α* and *γ* reflect an assumed ±5% variation in ***N***_**Ex**_, ***N***_**Th**_, and T. The two estimates for the enhancement, *∈*, show the variation when using the full set of residues with *N* = 165 and with the six proline residues of CypA excluded such that *N* = 159.

## Supporting Material

Document S1. Supporting Materials and Methods, seven figures, and one table

Document S2. Article plus Supporting Material
