# Supplementary material for: Characterization of Folding Cores in the Cyclophilin A-Cyclosporin A Complex
Source: Biophys J. 2015 Apr 7;108(7):1739–46. doi: 10.1016/j.bpj.2015.02.017 (PMC4390823; doi:10.1016/j.bpj.2015.02.017)
Supplement: Document S1. Supporting Materials and Methods, seven figures, and one table [file mmc1.pdf]

Supporting Material

Flexibility-based predictions of folding cores: characterization of the cyclophilin A – cyclosporin A complex

J. W. Heal, C. A. Blindauer, S. A. Wells, R. B. Freedman and R. A. Römer

1 Modeling hydrophobic tethers in FIRST

HPs are indirect, entropy-driven interactions (1) thought to contribute significantly to protein folding (2). HPs have not always been included in FIRST simulations, and their effect on modeling the flexibility and mobility of proteins is still not fully understood. In early papers using FIRST, HPs were not included in the bond network (3, 4), whereas more recently it is usual practice to include HPs and maintain them throughout rigidity dilution (5, 6) or to increase their number as E_{cut} is lowered (7, 8). In FIRST, HPs are modeled as flexible constraints, restricting the separation distance between atoms involved in the interaction, but not the angle between them. In this way, the interacting atoms are permitted to slip relative to each other (9, 10). HPs between carbon or sulfur atoms are typically included if the distance between these atoms is less than the sum of their van der Waals radii, r_v, plus a distance cutoff, D_{HP}. For carbon and sulfur, $r_v = 1.7$ Å and 1.8 Å respectively, and D_{HP} is typically set to 0.25 Å (6, 9, 10). These distances allow us to define the burial distance of a given atom (see below).

2 Effects of the modified bond network and the inclusion of mobility

Let us now define in detail how we distinguish buried and exposed residues. For each atom in the protein, a sphere of radius $r_v + r_w$, formed from 400 points, is drawn with the atom in the centre, where r_v is the van der Waals radius of the atom and $r_w = 1.4$ Å is that of a water molecule; for nitrogen, $r_v(N) = 1.5$ Å. The points forming the sphere are then individually checked for contact with the neighboring atoms. If any point on the sphere is not in contact with a neighbor then that is a potential solvent position and the atom is labeled as being exposed. An exposed atom has $R = 0$ Å. If an atom is not exposed, it is buried and its burial distance, R, is the shortest distance to an exposed atom. For a given conformer, we calculated R for each nitrogen atom in the amide backbone and used this as the value of R for the residue to which it belongs.

We measured the surface exposure of each amide nitrogen in the structure 1CWA, shown in Figure S1. For the small protein CypA, we find that the majority of the residues are somewhat exposed to the protein surface, with $R < r_v(N) = 1.5$ Å. Since we measure the burial of the amide nitrogen and the length of the N-H bond is 1.5 Å, if the H atom is exposed to the surface, then the amide nitrogen has $R \leq 1.5$ Å. In Figure S1 we also show a superposition of FIR_B along the protein backbone as taken from the rigidity dilution plot. We note that most of the residues that are highly buried within the protein structure ($R > 2.0$ Å) correspond to regions of the protein that are part of the folding core. This shows that the majority of those residues that are buried in the CypA-CsA complex are in fact rigid and buried.

3 Effect of ligand binding on folding cores

There are only small changes to the HDX folding core upon ligand binding, and these are not captured using rigidity analysis alone. Indeed, only seven residues are part of the HDX folding core for the complex but not for the unbound protein. Upon ligand removal, FIR_B changes by a single residue, which is not one of these seven, and FIR changes substantially and unexpectedly. These two folding cores solely derived using FIRST do not reflect the effect observed experimentally. We see a larger effect of ligand binding on the folding core predicted by FRO. Figure S2 shows how ligand binding affects the burial distance R. We calculated the average R for each residue from the final conformers of the FRODA simulations, and plotted the absolute difference, ΔR, between these values. Of the eleven residues for which $\Delta R > 0.5$ Å, five are binding site residues and none are further than two residues along the backbone from a binding site residue. We also calculated ΔR for each residue from FRODA simulations carried out at $E_{\text{cut}} = -0.5$, -1.0, -1.5 and -3.0 kcal/mol. In each case, the residues which are part of, or close to, the binding site have the largest ΔR (data not shown). The HDX folding core changes in sections of the protein more distant from the binding site, and so tracking the protein surface in FRODA does not capture this effect.

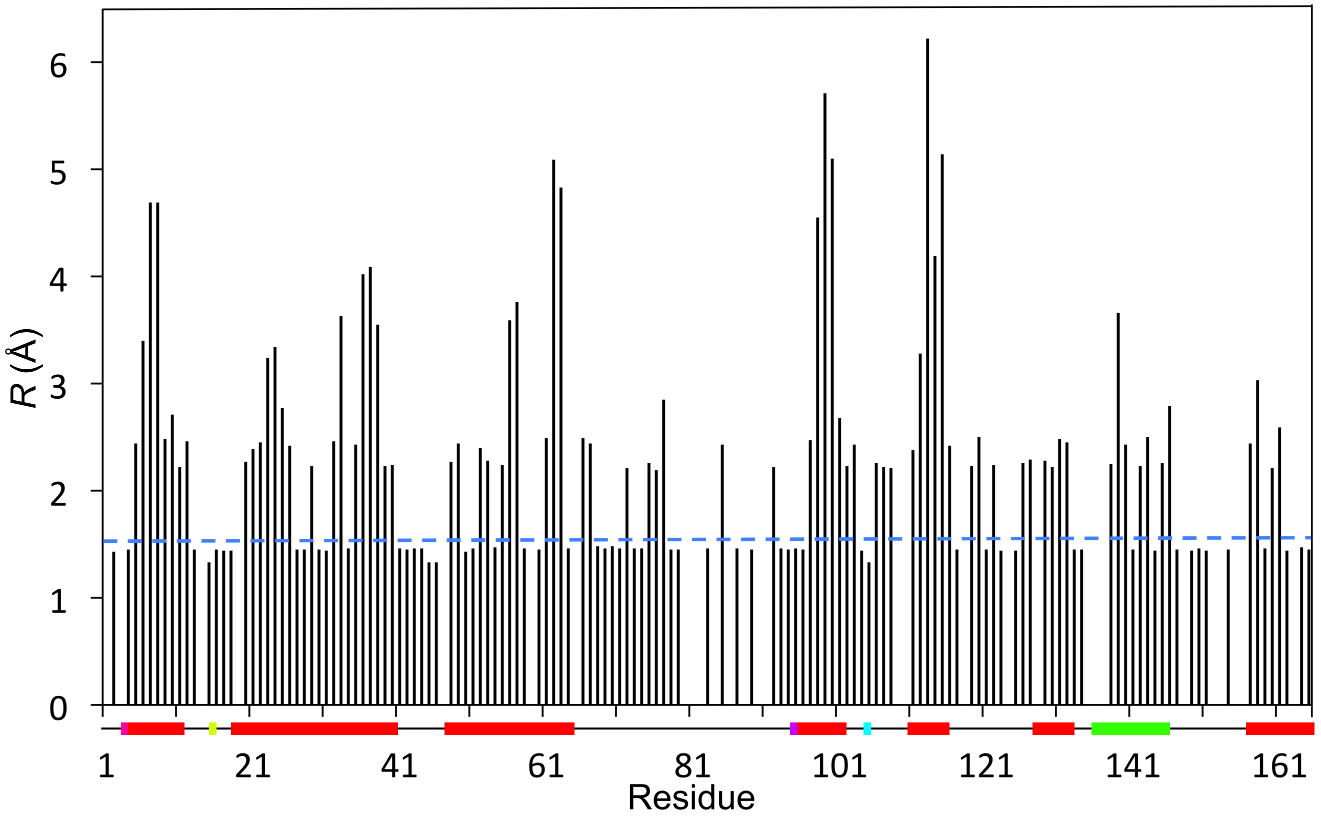

Figure S1: The burial distance R for each residue of CypA in the static structure of the CypA-CsA complex. The blue dashed line at $R = 1.5$ Å is used to separate exposed residues (below the line) from buried residues (above the line). Below the horizontal axis, we schematically show the extent of the FIR_B folding core along the residues via coloured regions. Those residues which are part of FIR_B are coloured red; other rigid residues are coloured otherwise. The thin horizontal black line denotes residues that are flexible.

Our overall computational strategy is based on using the results from HDX experiments as the benchmark against which theoretical results are compared. The measures α, γ and ϵ score most highly when the match of a theoretical folding core with the HDX result is perfect. However, the HDX data may contain errors themselves and this additional source of variation did not play a role in our judgements of the success of the computational approaches. Furthermore, for the CypA-CsA complex, the protein is large relative to the ligand and the ligand's effect on HDX is rather small. We expect that an application of our methods to a larger protein that exhibits a more significant conformational change upon ligand binding will also give a clearer change in the values of α, γ and ϵ.

4 Comparison with the COREX algorithm

The COREX algorithm has previously been used to predict the behaviour of proteins in HDX (11, 12). Five proteins, hen egg-white lysozyme, equine lysozyme, bovine pancreatic trypsin inhibitor, staphylococcal nuclease and turkey ovomucoid third domain were studied in (11), each without a ligand bound. Each of these proteins in the study is considered small by the authors (< 150 residues); CypA has 165 residues and so would not fit into this description. The study is different from ours in that it focuses on five proteins using one model, whereas we study one protein in the presence and absence of a ligand, using five different models. However, we can interpret their results in terms of α, specificity and γ, sensitivity. The α values for the five small proteins are 0.72, 0.70, 0.79, 0.77 and 0.65 respectively. Our α values for the unbound CypA range between 0.59 and 0.86, and between 0.66 and 0.81 for the CypA-CsA complex. In both cases, the highest α values are for the combined FIRST and FRODA approach. In terms

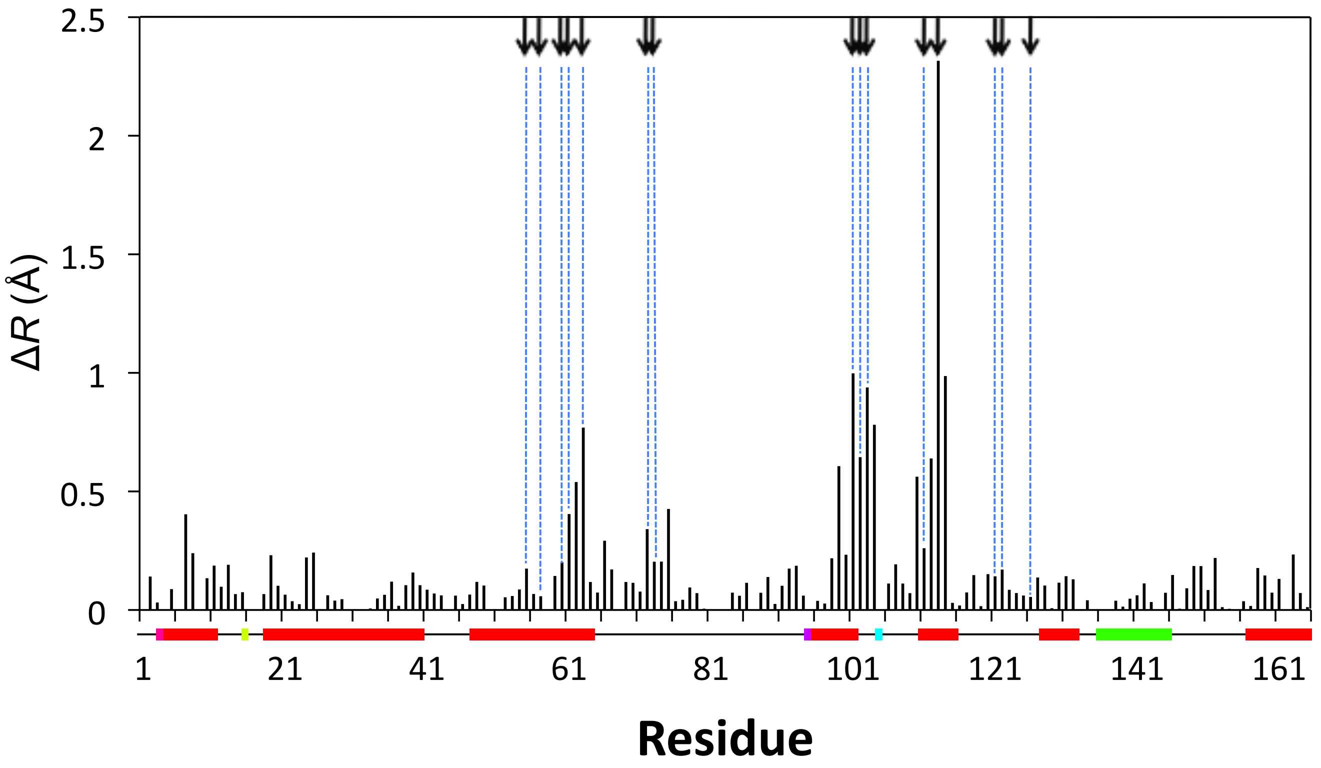

Figure S2: The difference in burial distance ΔR (black bars) between the CypA-CsA complex and the unbound protein as derived from the motion analysis via FRODA is plotted for each amino acid. The arrows and vertical dotted lines indicate the binding residues. As in Figure S1, FIR_B is also shown below the horizontal axis.

of specificity, γ values of 0.88, 0.85, 0.74, 0.88 and 0.90 are high for the five proteins in (11). For unbound CypA, our range of γ values, between 0.70 and 0.90 compares well, whereas for the CypA-CsA complex, values between 0.55 and 0.74 are comparatively poor. A further area of investigation would be to carry out a more comprehensive comparison between the combined FIRST–FRODA approach and that of the COREX algorithm. Such a study would need to include a range of protein sizes, a discussion of the computing power required and a consideration of the power of these algorithms to elucidate subtle binding effects such as those observed for the formation of the CypA-CsA complex.

5 Protein expression and purification

A derivative of the pQE-70 plasmid encoding for human CypA was expressed in *E. coli* (JM109, New England BioLabs) grown at 37°C in minimal medium containing 1 g/L $(^{15}NH_4)_2SO_4$ (Cambridge Isotope Laboratories). When the growth cultures reached an optical density of 0.5 at 600 nm, protein expression was induced with 1 mM isopropyl-β-D-thiogalactopyranoside (IPTG). Cells were harvested after overnight growth during which selection pressure was maintained by adding 0.1 mg/mL ampicilin. Cell pellets were resuspended in 20 mM 4-(2-hydroxyethyl)-1-piperazineethanesulfonic acid (HEPES) buffer at pH = 6.5 and then stored at −20°C. Frozen cells were thawed, sonicated and then centrifuged, after which the resulting supernatant was loaded onto a 10 mL Source 30S column (from GE Healthcare) for cation exchange using 20 mM HEPES buffer at pH = 6.5 and a concentration gradient of 0 − 150 mM NaCl. For NMR experiments, eluted fractions of CypA were dialysed overnight against 10 mM ammonium acetate buffer at pH = 6.5 before being concentrated and lyophilised.

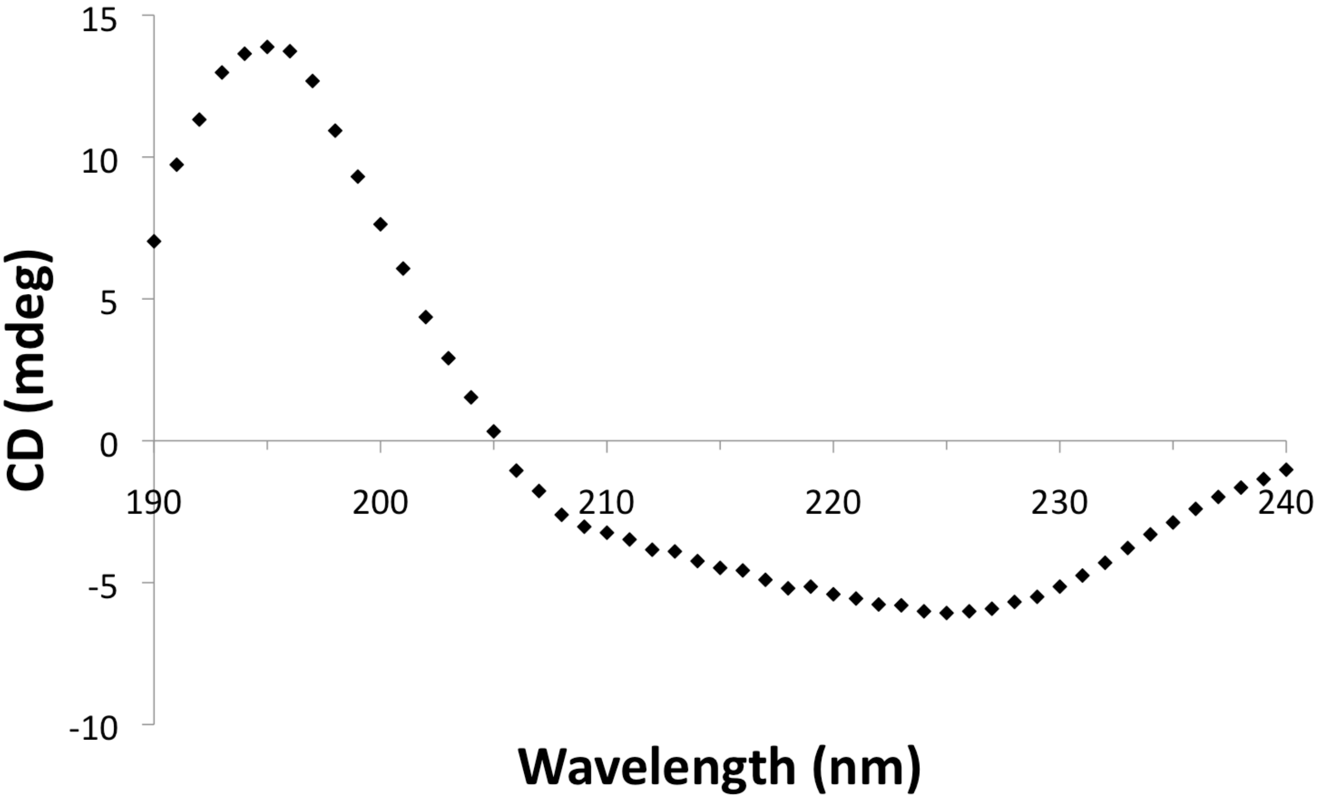

Figure S3: Far-UV CD spectrum of CypA at 25°C. The average CD signal from 16 scans is plotted at 1 nm intervals between 190 nm and 240 nm.

6 Circular dichroism spectroscopy

To estimate the secondary structure composition for purified CypA in order to verify that it was correctly folded, the far-UV CD spectrum of CypA was measured at 25°C using a Jasco J-815 CD spectropolarimeter. The resulting spectrum is shown in Figure S3. CD spectra were recorded for protein samples of 0.1 mg/mL in 5 mM sodium phosphate, pH 7.3 unless otherwise stated. Small volumes of concentrated purified protein were diluted in sodium phosphate buffer and the resulting concentration confirmed by measuring A_{280}. Data points were collected during far-UV scans between 180 nm and 260 nm at 1 nm intervals. A baseline spectrum was recorded in the same way for a sample containing buffer only. To generate Figure S3, 16 scans were collected at 100 nm/min in continuous scanning mode, and the average CD signal minus the average baseline signal was plotted. We show the data collected between 190 nm and 240 nm at 1 nm intervals. The data was analysed with Dichroweb, using the reference database SP175 to calculate the proportional secondary structure composition of CypA. Our protein sample was determined to be 18 % helix and 33 % sheet. We compared this with the results of the DSSP algorithm applied to five X-ray crystal structures in the PDB. The average composition of these structures was 13 % α-helix and 32 % β-sheet.

7 Fluorescence spectroscopy

When CsA binds to CypA, Trp121 becomes shielded from the solvent and its fluorescence increases as a result (13, 14). CsA was titrated into a solution of CypA and fluorescence spectroscopy was used to monitor the change in tryptophan fluorescence. The experiment was conducted using a Photon Technology International fluorimeter. A stock solution of CypA (65 μM) was diluted 1 mL in 50 mM TRIS buffer at pH 7.3 so that the final CypA concentration was 4.5 μM. CsA was stored in ethanol at a concentration of 1.0 mM. A stock solution of 0.1 mM CsA for the titration was made by diluting this ten-fold in TRIS buffer. Each titre consisted of 10 μL of CsA stock,

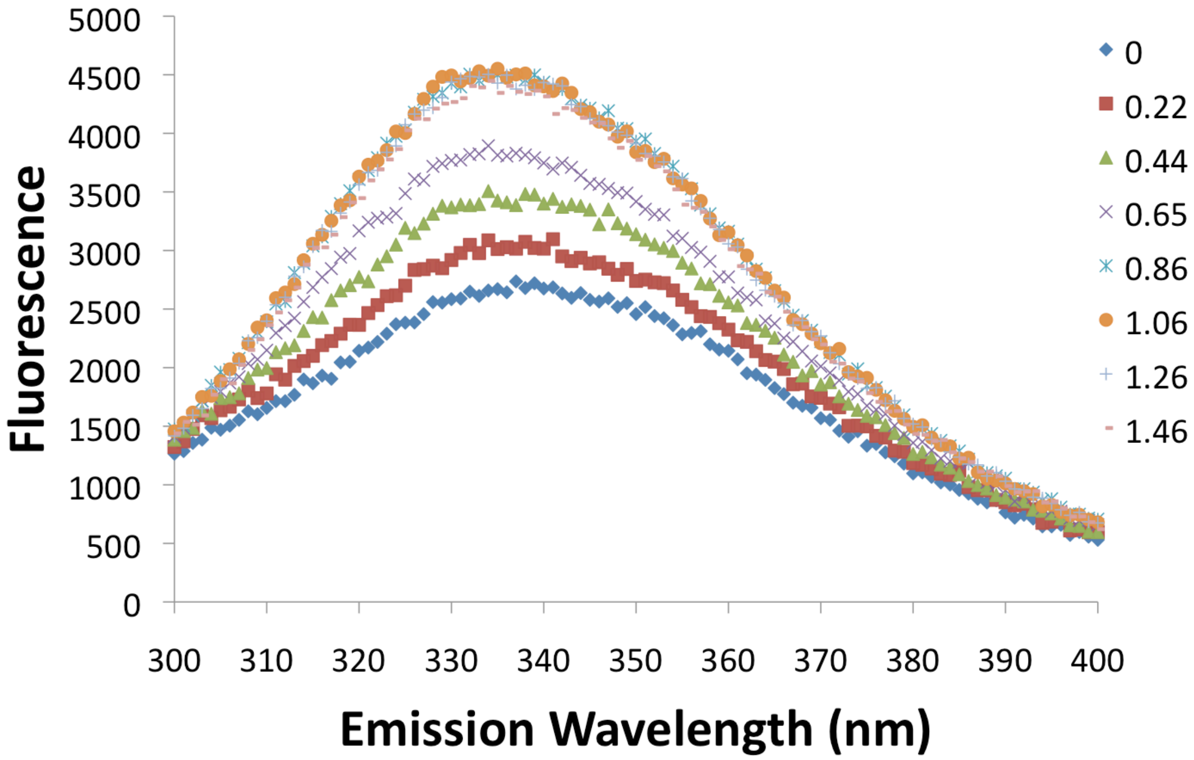

Figure S4: Fluorescence emission spectra of CypA with increasing CsA concentration. The key shows the concentration ratio [CsA]/[CypA]. Emission spectra were recorded at 1 nm intervals between 300 nm and 400 nm with excitation wavelength of 290 nm. The average emission value of three scans is plotted.

and so increased the total concentration of CsA in the fluorescence sample by approximately 1 μM. After each increment in [CsA] the sample was mixed using a pipette. The emission spectra were recorded at 1 nm intervals between 300 nm and 400 nm, using an excitation wavelength of 290 nm. The average fluorescence emission of three scans was recorded for each wavelength, using a slit width of 2 nm. Two baselines were recorded, for samples containing TRIS buffer only and 5 μM CsA in TRIS buffer. There was no significant difference between the baselines (data not shown). Baseline adjusted fluorescence emission spectra for CypA with various concentrations of CsA are shown in Figure S4. The key shows the concentration fraction [CsA]/[CypA]. We observe a steady increase in fluorescence with increasing [CsA] until [CsA]/[CypA] = 1. After this point is reached, adding more CsA does not enhance fluorescence.

8 NMR spectrum assignments

For sequential assignment, lyophilised protein was resuspended in NMR buffer containing 4.2 mM NaH_2PO_4, 15.8 mM Na_2HPO_4 and 150 mM NaCl at pH 6.5. 2D [^{1}H,^{15}N] HSQC and 3D [^{1}H, ^{15}N, ^{1}H] TOCSY-HSQC and NOESY-HSQC data were acquired on a Bruker AV III 600 spectrometer operating at 600.13 MHz for ^{1}H and 60.81 MHz for ^{15}N. 2D data were acquired with 16 scans, 2048 datapoints in F2 and 128 increments in F1, and Fourier transformed with 2048×512 datapoints over spectral widths of 16 ppm in the ^{1}H dimension (F2) and 42 ppm in the ^{15}N dimension (F1). 3D data were acquired with 8 or 16 scans and $2048 \times 40 \times 160$ datapoints in F3, F2 and F1, respectively, and transformed with $2048 \times 64 \times 512$ datapoints. Spectral widths were 16 ppm in the ^{1}H dimensions (F3, F1), and 38 ppm in the ^{15}N dimension (F2). Data were acquired and processed using TOPSPIN version 2.1 (Bruker) and analyzed using SPARKY version 3.1 (15). With this data, and the aid of previously published assignments for the CypA-CsA complex under different conditions (16, 17), we have assigned 147 of the 159 non-proline residues of the protein in its unbound state as well as in complex with CsA.

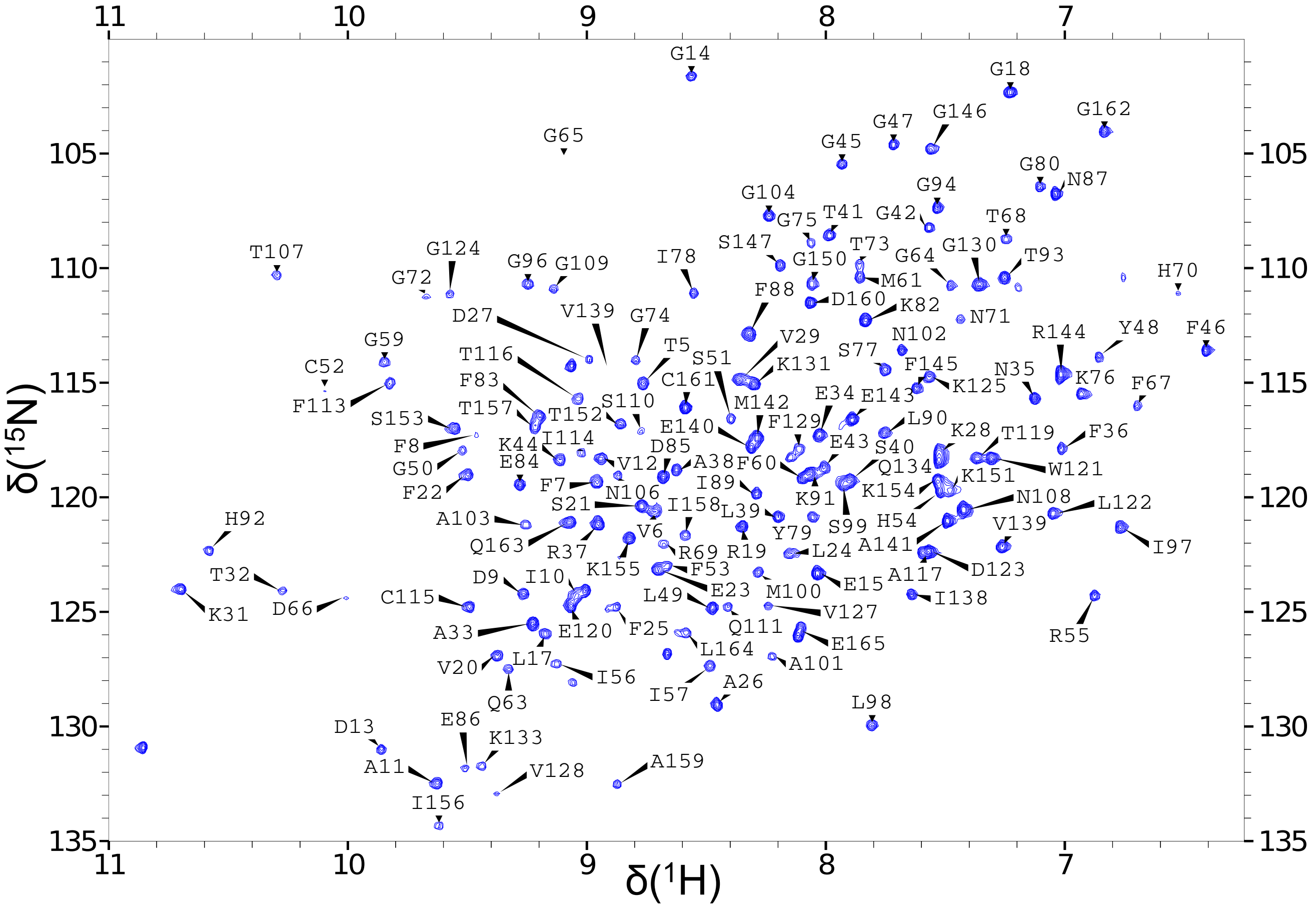

Figure S5: HSQC spectrum of the CypA-CsA complex. Blue contour lines show signal intensity, and assigned backbone N-H cross peaks are labeled. Chemical shift frequencies $\delta(^{1}H)$ and $\delta(^{15}N)$ are given in parts per million (ppm).

The unassigned residues include residues 1 – 4, which make up the flexible N-terminus. In Figure S5, we show the assigned HSQC spectrum for the CypA-CsA complex. Full lists of backbone N-H assignments for this spectrum and for the unbound protein are given in Section 10.

9 HDX experiments

2D [^{1}H,^{15}N] HSQC data for the HDX experiments were acquired on a Bruker AV II 700 spectrometer, equipped with a TCI cryoprobe, operating at 700.24 MHz for ^{1}H and 70.96 for ^{15}N. The lyophilised protein was resuspended in NMR buffer (4.2 mM NaH_2PO_4, 15.8 mM Na_2HPO_4 and 150 mM NaCl at pH 6.5) as above, but made up in 99.9% D_2O (Sigma-Aldrich). For the CypA-CsA sample, each spectrum was acquired with four scans, 2048 points in F2, and 64 increments in F1, and Fourier transformed with 2048 $\times$ 256 datapoints. The spectral widths were 16 ppm and 42 ppm in the ^{1}H and ^{15}N dimensions, respectively. In all cases, chemical shifts δ were referenced to the residual HDO peak (18).

Figure S6 shows the HSQC spectrum of the CypA-CsA complex alongside spectra recorded 10, 110 and 4270 minutes (71 hours and 10 minutes) after initiating HDX. For unbound CypA, we increased the number of scans recorded for each spectrum from 4 to 16 in response to a lower yield in order to maximise the signal to noise ratio. The earliest usable HSQC spectrum was completed 53 minutes after adding D_2O to the protein sample, and the subsequent spectra were therefore recorded less frequently than with the CypA-CsA experiments. Figure S7

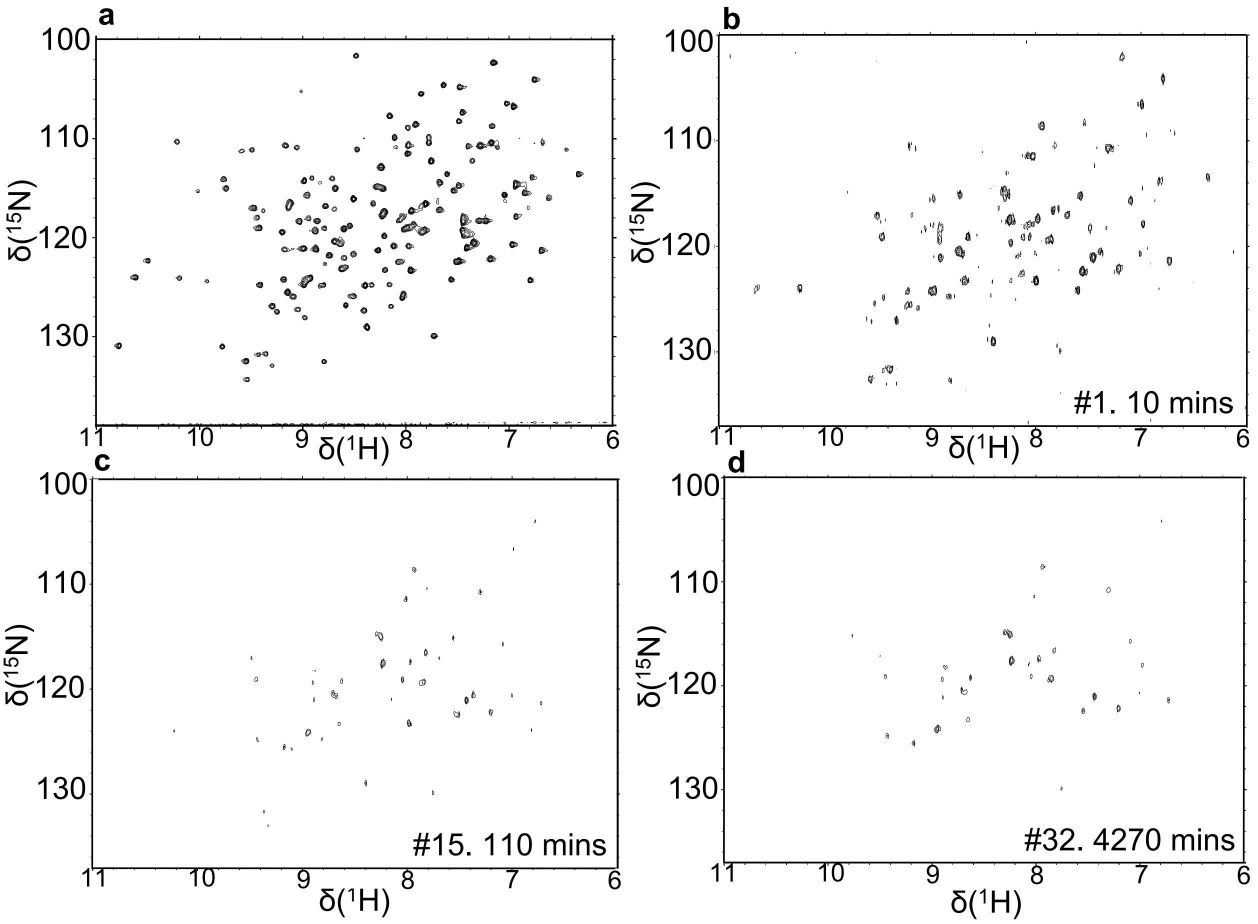

Figure S6: (a) HSQC spectrum of the CypA-CsA complex as in Figure S5. Following initiation of HDX, spectra were recorded after an elapsed time of, among others, (b) 10 minutes, (c) 110 minutes and (d) 4270 minutes. In each spectrum, N-H cross peaks are shown as contour lines, representing signal intensity. Chemical shifts $\delta(^1H)$ and $\delta(^{15}N)$ are given in ppm.

shows the full HSQC spectrum, along with spectra taken at different time intervals following the initiation of HDX. Spectra numbers 1, 4 and 22 are shown, recorded after 53, 113 and 4349 minutes respectively.

10 N-H assignment tables

Table S1 gives the chemical shift assignments for each residue of unbound protein as well as the CypA-CsA complex.

Table S1: Chemical shift assignment of CypA backbone ^{15}N-^1H pairs, for the unbound protein as well as the CypA-CsA complex. Chemical shifts are given in ppm. Blank cells represent unassigned signals.

Residue	CypA only		CypA-CsA		Residue	CypA only		CypA-CsA	
	^1H	^{15}N	^1H	^{15}N		^1H	^{15}N	^1H	^{15}N
Thr5	8.78	115.02	8.76	115.03	Glu86	9.45	131.63	9.51	131.82
Val6	8.74	120.4	8.71	120.65	Asn87	7.05	106.84	7.04	106.78
Phe7	8.95	119.23	8.96	119.34	Phe88	8.33	113.02	8.32	112.9
Phe8	9.55	117.03	9.56	117.01	Ile89	8.3	119.89	8.29	119.85
Asp9	9.27	124.24	9.27	124.22	Leu90	7.74	117.28	7.75	117.2

Residue	CypA only		CypA-CsA		Residue	CypA only		CypA-CsA	
	^{1}H	^{15}N	^{1}H	^{15}N		^{1}H	^{15}N	^{1}H	^{15}N
Ile10	9.03	124.31	9.04	124.27	Lys91	8.07	119.05	8.07	119
Ala11	9.62	132.57	9.63	132.48	His92	10.7	122.57	10.58	122.33
Val12	8.93	118.47	8.94	118.31	Thr93	7.26	110.48	7.25	110.42
Asp13	9.85	131.09	9.86	131	Gly94	7.48	107.31	7.53	107.37
Gly14	8.55	101.75	8.56	101.63	Pro95	N/A	N/A	N/A	N/A
Glu15	8.04	123.34	8.03	123.33	Gly96	9.27	110.31	9.25	110.7
Pro16	N/A	N/A	N/A	N/A	Ile97	6.76	121.49	6.77	121.31
Leu17	9.2	126.13	9.17	125.96	Leu98	7.87	128.89	7.81	129.95
Gly18	7.24	102.52	7.23	102.33	Ser99	8.28	118.81	7.94	119.4
Arg19	8.34	121.29	8.35	121.28	Met100			8.28	123.27
Val20	9.37	126.94	9.38	126.92	Ala101	7.99	125.97	8.22	126.95
Ser21	8.76	120.36	8.77	120.39	Asn102	8.11	113.4	7.68	113.59
Phe22	9.51	119.11	9.5	119.02	Ala103	8.77	123.37	9.18	121.03
Glu23	8.74	123.23	8.7	123.15	Gly104	8.18	109.38	8.24	107.71
Leu24	8.17	122.5	8.14	122.45	Pro105	N/A	N/A	N/A	N/A
Phe25	8.82	124.75	8.87	124.77	Asn106	8.86	119.13	8.87	119.05
Ala26	8.43	129	8.46	129.05	Thr107	10.21	110.39	10.3	110.31
Asp27	8.97	114.09	8.99	113.99	Asn108	7.37	120.61	7.42	120.56
Lys28	7.53	118.08	7.53	118	Gly109	9.15	110.83	9.14	110.91
Val29	8.36	114.64	8.36	114.86	Ser110	8.76	117.15	8.77	117.12
Pro30	N/A	N/A	N/A	N/A	Gln111	8.37	124.52	8.41	124.79
Lys31	10.66	123.84	10.7	124.02	Phe112	8.05	117.86	8.15	118.29
Thr32	10.28	124.06	10.27	124.08	Phe113	9.8	116.5	9.83	115.02
Ala33	9.28	125.67	9.23	125.52	Ile114			9.02	118.07
Glu34	8.02	117.28	8.03	117.29	Cys115	9.59	125.52	9.49	124.78
Asn35	7.12	115.69	7.13	115.69	Thr116	8.95	115.72	9.04	115.71
Phe36	7.01	117.92	7.02	117.89	Ala117	7.61	122.32	7.58	122.41
Arg37	8.94	121.1	8.95	121.15	Lys118	8.69	119.87		
Ala38	8.69	119.16	8.62	118.81	Thr119	7.61	120.21	7.37	118.3
Leu39	8.17	120.78	8.2	120.86	Glu120	9.09	124.63	9.06	124.65
Ser40	7.89	119.28	7.89	119.3	Trp121	7.25	117.92	7.3	118.3
Thr41	7.97	108.49	7.98	108.56	Leu122	7.01	120.05	7.05	120.72
Gly42	7.57	108.4	7.57	108.24	Asp123	7.59	122.24	7.56	122.37
Glu43	8.01	118.7	8.01	118.7	Gly124	9.53	111.33	9.57	111.14
Lys44	9.1	118.55	9.11	118.38	Lys125	7.74	115.68	7.57	114.75
Gly45	7.93	105.59	7.93	105.48	His126	7.61	120.21		
Phe46	6.41	113.75	6.41	113.59	Val127			8.24	124.74
Gly47	7.75	104.7	7.72	104.61	Val128	9.47	133.07	9.38	132.94
Tyr48	6.88	113.79	6.86	113.89	Phe129	8.1	117.87	8.11	117.89
Lys49	8.47	124.92	8.48	124.84	Gly130	7.36	110.79	7.36	110.73
Gly50	9.48	117.9	9.52	117.97	Lys131	8.34	115.36	8.3	115.06
Ser51	8.38	116.5	8.4	116.58	Val132	9.03	124.26		
Cys52	10.01	115.3	10.1	115.31	Lys133	9.47	131.87	9.44	131.73
Phe53	8.69	123.05	8.66	122.99	Glu134	7.53	118.49	7.53	118.5
His54	7.56	119.88	7.52	119.7	Gly135				
Arg55	7.04	123.14	6.87	124.3	Met136	8.85	122.66	8.86	122.66
Ile56	9.19	126.48	9.13	127.27	Asn137	9.05	114.4	8.91	114.42
Ile57	8.54	123.02	8.49	127.36	Ile138	7.64	124.28	7.64	124.24
Pro58	N/A	N/A	N/A	N/A	Val139	7.24	121.97	7.26	122.16
Gly59	9.73	114.24	9.85	114.11	Glu140	8.26	117.69	8.31	117.82
Phe60	8.17	119.37	8.1	119.19	Ala141	7.49	121.13	7.49	121.04
Met61	8.08	111.2	7.86	110.41	Met142	8.29	117.3	8.29	117.41

Residue	CypA only		CypA-CsA		Residue	CypA only		CypA-CsA	
	^{1}H	^{15}N	^{1}H	^{15}N		^{1}H	^{15}N	^{1}H	^{15}N
Cys62	8.47	114.88			Glu143	7.82	116.36	7.89	116.6
Gln63	8.72	127.99	9.33	127.5	Arg144	7.02	114.61	7.02	114.63
Gly64	7.36	110.79	7.48	110.77	Phe145	7.61	115.42	7.62	115.25
Gly65	9.36	106	9.1	105.22	Gly146	7.5	104.75	7.56	104.82
Asp66	9.96	124.06	10.01	124.4	Ser147	8.2	110.11	8.19	109.89
Phe67	6.62	116.04	6.69	116	Arg148				
Thr68	7.28	109.02	7.25	108.73	Asn149				
Arg69	8.65	122.09	8.68	122.05	Gly150	8.04	110.22	8.06	110.65
His70			6.53	111.11	Lys151	7.53	119.89	7.47	119.8
Asn71	7.49	112.49	7.44	112.24	Thr152	8.85	116.65	8.86	116.81
Gly72	9.66	110.6	9.67	111.25	Ser153	9.41	117.05	9.46	117.28
Thr73	7.93	112.2	7.86	109.83	Lys154	7.53	119.37	7.53	119.27
Gly74	8.71	114.04	8.8	114.03	Lys155	8.78	121.73	8.82	121.79
Gly75	8.11	109.15	8.06	108.9	Ile156	9.61	134.42	9.62	134.33
Lys76	6.97	115.71	6.93	115.5	Thr157	9.24	117.07	9.22	116.94
Ser77	7.79	114.41	7.75	114.44	Ile158	8.58	121.7	8.59	121.66
Ile78	8.55	111.24	8.55	111.1	Ala159	8.87	132.53	8.87	132.53
Tyr79	8.03	120.83	8.06	120.86	Asp160	8.06	111.55	8.06	111.51
Gly80	7.1	106.64	7.1	106.46	Cys161	8.58	116.13	8.59	116.09
Glu81					Gly162	6.84	104.18	6.84	104.04
Lys82	7.86	112.72	7.83	112.28	Gln163	9.05	121.01	9.08	121.11
Phe83	9.17	116.64	9.2	116.47	Leu164	8.59	126.15	8.59	125.92
Glu84	9.23	119.54	9.28	119.46	Glu165	8.13	126.36	8.12	126.08
Asp85	8.59	118.9	8.62	118.81					

References

[1] Folch, B., M. Rooman, and Y. Dechouck, 2008. Thermostability of salt bridges versus hydrophobic interactions in proteins probed by statistical potentials. J. Chem. Inf. Model. 48:119–127.

[2] Dill, K. A., 1990. Dominant forces in protein folding. Biochemistry 29:7133–7155.

[3] Thorpe, M. F., B. M. Hespenheide, Y. Yang, and L. A. Kuhn, 2000. Flexibility and Critical Hydrogen Bonds in Cytochrome c. Pac. Symp. Biocomput. 191–202.

[4] Jacobs, D., A. Rader, L. Kuhn, and M. Thorpe, 2001. Protein flexibility predictions using graph theory. Prot: Struct. Func. Gen. 44:150–165.

[5] Zavodszky, M. I., M. Lei, M. F. Thorpe, A. R. Day, and L. A. Kuhn, 2004. Modeling Correlated Main-Chain Motions in Proteins for Flexible Molecular Recognition. Prot: Struct. Func. Gen. 57:243–261.

[6] Radestock, S., and H. Gohlke, 2008. Exploiting the Link between Protein Rigidity and Thermostability for Data-Driven Protein Engineering. Eng. Life Sci. 8:507–522.

[7] Rathi, P. C., S. Radestock, and H. Gohlke, 2012. Thermostabilizing mutations preferentially occur at structural weak spots with a high mutation ratio. J. Biotechnol. 159:135–144.

[8] Pfleger, C., P. C. Rathi, D. L. Klein, S. Radestock, and H. Gohlke, 2013. Constraint Network Analysis (CNA): a Python software package for effciently linking biomacromolecular structure, flexibility, (thermo-)stability, and function. J. Chem. Inf. Model. 53:1007–1015.

[9] Hespenheide, B. M., A. J. Rader, M. F. Thorpe, and L. A. Kuhn, 2002. Identifying Protein Folding Cores: Observing the Evolution of Rigid and Flexible Regions during Unfolding. J. Mol. Graph. & Model. 21:195–207.

[10] Gohlke, H., L. A. Kuhn, and D. A. Case, 2004. Change in protein flexibility upon complex formation: analysis of ras-raf using molecular dynamics and a molecular framework approach. Prot: Struct. Func. Bioinf. 56:332–337.

[11] Hilser, V. J., and E. Freire, 1996. Structure-based Calculation of the Equilibrium Folding Pathway of Proteins. Correlation with Hydrogen Exchange Protection Factors. J. Mol. Biol. 262:756–772.

[12] Luque, I., and E. Freire, 2000. Structural Stability of Binding Sites: Consequences for Binding Affinity and Allosteric Effects. Prot: Struct. Func. Gen. Suppl. 4:63–71.

[13] Liu, J., M. W. Albers, C. Chen, S. L. Schreiber, and C. T. Walsh, 1990. Cloning, expression, and purification of human cyclophilin in Escherichia coli and assessment of the catalytic role of cysteines by site-directed mutagenesis. P. Natl. A. Sci. USA 87:2304–2308.

[14] Husi, H., and M. G. M. Zurini, 1994. Comparative binding studies of cyclophilins to cycosporin A and derivatives by

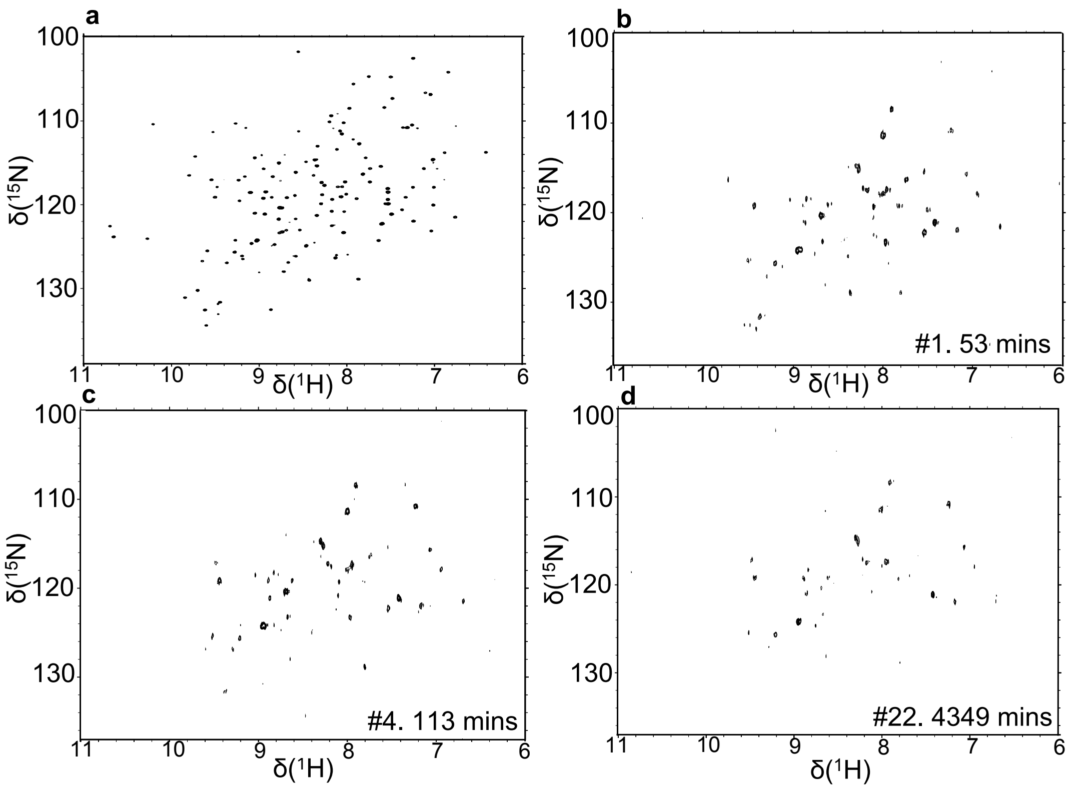

Figure S7: (a) HSQC spectrum of unbound CypA. HSQC spectra are also shown at different time intervals following initiation of HDX. These are (b) 53 minutes, (c) 113 minutes and (d) 4349 minutes.

fluorescence measurements. Anal. Biochem. 222:251–255.

[15] Goddard, T. D., and D. G. Kneller. SPARKY 3. University of California, San Francisco.

[16] Neri, P., R. Meadows, G. Gemmecker, E. Olejniczak, D. Nettesheim, T. Logan, R. Simmer, R. Helfrich, T. Holzman, J. Severin, and S. Fesik, 1991. ^{1}H, ^{13}C and ^{15}N backbone assignments of cyclophilin when bound to cyclosporin A (CsA) and preliminary structural characterization of the CsA binding site. FEBS J. 294:81–88.

[17] Ottiger, M., O. Zerbe, P. Güntert, and K. Wütrich, 1997. The NMR solution conformation of unligated human cyclophilin A. J. Mol. Biol. 272:64–81.

[18] Wishart, D. S., C. G. Bigam, J. Yao, F. Abildgaard, H. J. Dyson, E. Oldfield, J. L. Markley, and B. D. Sykes, 1995. ^{1}H, ^{13}C and ^{15}N chemical shift referencing in biomolecular NMR. J. Biomol. NMR 6:135–140.
